# Association Between Lens Thickness and SRK/T Refractive Prediction Error After Cataract Surgery: A Single‐Center Retrospective Cohort Study

**DOI:** 10.1155/joph/4944226

**Published:** 2026-09-22

**Authors:** Tao Liu, Haifeng Jiang, Yanxia Tong, Zhanpeng Zhu, Lihan Luo, Yong Wang

**Affiliations:** ^1^ Zhoukou Aier Eye Hospital, Zhoukou, Henan, China; ^2^ Aier Eye Hospital of Wuhan University (Wuhan Aier Eye Hospital), Wuhan, Hubei, China

**Keywords:** cataract, effective lens position, lens thickness, refractive error

## Abstract

**Purpose:**

To evaluate the association between lens thickness (LT) and SRK/T refractive prediction error following cataract surgery and to analyze its underlying mechanism.

**Methods:**

This retrospective cohort study collected data from patients who underwent surgical treatment for age‐related cataracts between January 2025 and June 2025. Patients were stratified into three groups based on LT: Group 1 (< 4.0 mm), Group 2 (4.0 ≤ LT < 5.0 mm), and Group 3 (≥ 5.0 mm). Intraocular lens (IOL) power was calculated using the SRK/T formula. Manifest refraction results were collected at ≥ 4 weeks postoperatively. Accuracy of IOL power calculation was analyzed using prediction error (PE), mean absolute error (MAE), median absolute error (MedAE), and the proportions of PE within ±0.25 D, ±0.50 D, and ±1.00 D. Correlations between LT and effective lens position (ELP) as well as postoperative refractive error were assessed.

**Results:**

A total of 431 patients (431 eyes) were included, with a mean LT of 4.50 ± 0.58 mm. The overall mean PE was −0.08 ± 0.44 D, and the mean ELP_error_ was −0.04 ± 0.43 mm. Statistically significant differences were observed across the three LT groups for both PE (*F* = 25.742, *p* < 0.001) and ELP_error_ (*F* = 10.838, *p* < 0.001). ANCOVA was further performed to adjust for age, AL, ACD, and WTW. After adjustment, the LT group effect on PE remained significant (*F* = 14.960, *p* < 0.001, partial *η* ^2^ = 0.066), with adjusted marginal means of −0.260 D, −0.118 D, and 0.165 D for Groups 1–3, respectively, and Bonferroni‐corrected pairwise comparisons confirming significant differences across all three groups (all *p* < 0.05). Group 3 (LT ≥ 5.0 mm) exhibited a positive mean PE (0.16 ± 0.45 D), whereas Groups 1 and 2 showed negative PEs. The MAE was ranked from lowest to highest as follows: Group 2 (0.33 D) < Group 3 (0.37 D) < Group 1 (0.40 D). Group 2 (4.0 ≤ LT < 5.0 mm) achieved the lowest MedAE (0.29 D), the highest proportion of PE within ±0.50 D (78.3%), and 100.0% within ±1.00 D. Significant positive linear correlations were observed between LT and PE (*r* = 0.331, *p* < 0.001) and between LT and ELP_error_ (*r* = 0.226, *p* < 0.001). Multivariate linear regression identified LT as the strongest predictor of PE (*β* = 0.490, *p* < 0.001), followed by ACD (*β* = 0.217, *p* = 0.012) and WTW (*β* = 0.130, *p* = 0.031), with the model explaining 21.2% of the variance in PE.

**Conclusion:**

Increased deviation of LT from the mean results in larger ELP prediction errors and greater postoperative refractive error. Thin‐lens eyes tended to be myopic, while thick‐lens eyes tended to be hyperopic.

## 1. Introduction

Over 30 million cataract surgeries are performed globally each year, with postoperative refractive error representing a core determinant of visual satisfaction among patients [1]. Even minor refractive deviations may induce symptoms of blurred vision and visual disturbances, thereby compromising surgical outcomes [2]. Clinical evidence indicates that approximately 30% of patients exhibit postoperative refractive errors exceeding ±0.5 diopters following cataract surgery [3]. Biometric parameters of the eye demonstrate significant correlations with refractive outcomes after cataract surgery. Previous investigations have predominantly focused on the associations between axial length (AL) and mean keratometry (*K*_*m*_) with refractive errors, while other biometric parameters remain underexplored [4].

As a fundamental anatomical parameter, lens thickness (LT) plays a critical role in maintaining the stability of the ocular refractive system [5]. Research has confirmed that preoperative LT and anterior chamber depth (ACD) constitute key determinants influencing the effective lens position (ELP) following cataract surgery, which subsequently compromises the accuracy of intraocular lens (IOL) power calculation formulas [6]. Particularly, conventional formulas based on “standard eye” models often fail to accommodate anatomical variations in eyes with abnormal LT [7]. Clinical observations reveal that patients with high myopia typically present with lens thinning, while hyperopic patients exhibit lens thickening. Moreover, cataract patients with concurrent lens subluxation or spherophakia demonstrate heterogeneous or pronounced LT abnormalities. These populations consistently show significantly higher rates of postoperative refractive errors compared to the general patient cohort [8–10].

Current evidence regarding the correlation between LT and postoperative refractive errors remains controversial. Melles et al. reported a positive correlation between prediction error (PE) and LT [11], whereas Chen et al. argued that the relationship between LT and PE varied across different AL subgroups [12]. Critical questions regarding the mechanistic pathways through which LT modulates postoperative refractive status, potential differences in refractive error profiles among distinct LT abnormality types, and threshold effects of LT deviations remain unresolved. Systematic mechanistic analyses addressing these issues are notably lacking in contemporary literature. Accordingly, this study aims to systematically evaluate the association between LT and postoperative refractive errors and to investigate the predictive value of LT in refractive outcomes following cataract surgery, thereby providing clinical evidence for optimizing IOL power calculation strategies and minimizing postoperative refractive errors.

## 2. Methods

### 2.1. Study Population

This retrospective cohort study analyzed data from 431 patients (431 eyes) who underwent phacoemulsification combined with implantation of nontoric IOL at Aier Eye Hospital of Wuhan University between January 2025 and June 2025. To avoid data duplication, only one eye per patient was included (when both eyes met inclusion criteria, eye selection was randomized). The study was approved by the Ethics Committee of Aier Eye Hospital of Wuhan University, and all research procedures adhered to the tenets of the Declaration of Helsinki (Approval NO: 2024IRBKY112507).

Inclusion criteria comprised (1) patients aged ≥ 40 years diagnosed with age‐related cataract; (2) successful phacoemulsification combined with nontoric IOL implantation; and (3) postoperative best‐corrected visual acuity (BCVA) ≥ 0.5.

Grouping criteria: Patients were categorized according to LT values: Group 1 (< 4.0 mm), Group 2 (4.0 ≤ LT < 5.0 mm), and Group 3 (≥ 5.0 mm).

Exclusion criteria included (1) prior ocular surgeries (e.g., corneal transplantation, refractive surgery, pterygium excision, or vitrectomy in the study eye, except for uncomplicated cataract surgery); (2) lens subluxation/dislocation; (3) IOL suspension or ciliary sulcus IOL implantation; (4) corneal disorders or corneal scars affecting refractometry; and (5) congenital cataracts or dense cataracts preventing accurate biometric measurements.

The IOL constants included in this study ranged from 118.0 to 119.5. The IOL models comprised monofocal IOLs (Alcon: AcrySof SN60WF; HumanOptics: ASPIRA‐aA, ASPIRA‐aAY; Johnson & Johnson: DIB00, ICB00, ZCB00; Lenstec: SBL; etc.), bifocal IOLs (Alcon: AcrySof SN6AD1; ZEISS: AT LISA 809MP; Johnson & Johnson: ZMB00; Lenstec: SBL‐2, SBL‐3; Teleon: LS‐313 MF15; etc.), trifocal IOLs (Alcon: AcrySof PanOptix TFNT00, Clareon PanOptix CNWTT0; ZEISS: AT LISA tri 839MP), and extended depth of focus (EDOF) IOLs (Alcon: AcrySof IQ Vivity DFT015; Johnson & Johnson: ZXR00, ZFR00V) from multiple IOL manufacturers. For all included IOLs, the optical constants were optimized values obtained from the ULIB website. For IOL models not listed on ULIB, the constants were provided by the manufacturers. For monofocal IOLs, the IOL power was based on the SRK/T formula calculation; for other IOL types, the power was determined with reference to both the Barrett Universal II and SRK/T formulas.

### 2.2. Preoperative Assessment

All patients underwent documentation of age, sex, ocular laterality, IOL model used during surgery, and actual IOL diopter power. Preoperative ocular biometric measurements were obtained using the IOL Master 700 (Carl Zeiss, Germany), including AL, LT, ACD, *K*_*m*_, horizontal corneal diameter (white‐to‐white, WTW), central corneal thickness (CCT), and IOL constants integrated into the IOL Master 700 software. All measurements were performed by a single experienced ophthalmologist following a standardized protocol. Additionally, manifest refraction results from 4 or more weeks postoperatively were collected. In cases where multiple preoperative refraction records were available for a patient; the refraction result farthest in time from the surgical date was retained for analysis.

### 2.3. Data Collection

Patients were followed up for 4–24 weeks postoperatively, and manifest refraction was performed at least 4 weeks after surgery to determine the actual postoperative refractive status. The refraction results were then converted to spherical equivalent (SE) using the formula: SE = S + C/2 (where S represents the spherical diopter and C represents the cylindrical diopter). The following parameters were calculated and recorded:

Prediction Error (PE): PE = Postoperative manifest refraction SE − Predicted postoperative SE. This metric assesses whether a formula systematically overestimates or underestimates IOL power. The value not only indicates the magnitude of deviation but also its direction. A more negative PE signifies a postoperative refractive outcome closer to myopia relative to the prediction, whereas a more positive PE indicates a hyperopic shift.

Mean absolute error (MAE): The arithmetic mean of absolute PE values, reflecting the overall predictive accuracy of the model.

Median absolute error (MedAE): The median of absolute PE values, representing the central tendency of prediction errors.

Smaller MAE and MedAE values indicate higher predictive accuracy and better model performance. Additionally, the percentages of PE within ±0.25 D, ±0.50 D, and ±1.0 D were calculated.

### 2.4. Calculation of Effective Lens Position Error (ELP_error_)

The predicted effective lens position (ELP_predict_), true effective lens position (ELP_true_), and ELP_error_ were calculated according to the SRK/T formula [7]. The SRK/T formula is a thin lens vergence formula that remains one of the most widely used IOL power calculation formulas in clinical practice worldwide, with a well‐characterized computational equation and a transparent mechanism for ELP estimation.

ELP_predict_: Calculated using Formula 1 based on preoperative AL, K_m_, and IOL constants.

ELP_true_: Calculated using Formula 2 based on preoperative AL, K_m_, postoperative SE, and IOL diopter power.

ELP_error_ = ELP_true_ − ELP_predict_; a positive value indicates ELP_true_ > ELP_predict_, while a negative value indicates ELP_true_ < ELP_predict_.

Formula 1. Calculation of ELP_predict_

Formula 1.1:

(1) Cw=−5.410.58412+×AL ,−3.4461.7160.0237+×AL−×AL2 , AL≤24.2mmAL>24.2mm.

Formula 1.2:

(2) ELPpredict=r−r2−Cw24r , , r≥Cw2r<Cw2+0.6246768.7473.336×Aconst−−.

In the formula, r represents the corneal radius of curvature, defined as *r* = ((1.3375 − 1)/*K*_*m*_) × 1000; A_const_ denotes the IOL constant; and AL indicates the patient’s preoperative AL.

Formula 2. Back‐calculation of ELP_true_

Formula 2.1:

(3) LOPT=AL+0.656960.02029−×AL.

Formula 2.2:

(4) REFTGT=11/na/na/na/LOPT−ELPtrue−IOLamet+ELPtrue−ncm1/r+V.

REFTGT = target refraction (in diopters, D); IOLamet = IOL power (in diopters, D); *r* = corneal curvature radius (in millimeters, mm), calculated as: *r* = ((1.3375 − 1)/*K*_*m*_) × 1000; AL = preoperative axial length (in millimeters, mm); *V* = vertex distance = 0.012 m; *n*_*a*_ = 1336; *n*_*c*_ *m*1 = 333.

### 2.5. Statistical Methods

Statistical analyses were performed using IBM SPSS Statistics for Windows, Version 25.0 (IBM Corp, Armonk, NY, USA). *p* value < 0.05 was considered statistically significant. All normally distributed quantitative data were presented as mean ± standard deviation (SD), while non‐normally distributed quantitative data were expressed as median [interquartile range (IQR)]. Multivariate linear regression analysis was conducted with postoperative refractive PE as the dependent variable, and seven preoperative parameters (age, AL, ACD, LT, *K*_*m*_, WTW, and CCT) as independent variables. Standardized regression coefficients (*β* values) were calculated to evaluate the relative importance of each predictor. For the analysis of relationships between LT, PE, and ELP_error_, one‐way analysis of variance (ANOVA), independent samples *t*‐test, and Pearson correlation analysis were employed. Furthermore, to eliminate the potential confounding effects of baseline differences across LT groups, analysis of covariance (ANCOVA) was further employed. Correlation strength was interpreted as weak correlation (−0.3 ≤ *r* < 0 or 0 < *r* ≤ 0.3), moderate correlation (−0.5 ≤ *r* < −0.3 or 0.3 < *r* ≤ 0.5), and strong correlation (*r* < −0.5 or *r* > 0.5). Data visualization was performed using a web‐based tool (https://cnsknowall.com/#/HomePage).

## 3. Results

### 3.1. Demography

This study enrolled 431 cataract patients (431 eyes), including 177 males (41.07%) and 254 females (58.93%), with 247 right eyes (57.31%) and 184 left eyes (42.69%). Comparative analysis of baseline characteristics revealed statistically significant differences between groups for age, AL, ACD, LT, WTW, IOL power, and ELP_error_ parameters (*p* < 0.05). No statistically significant differences were observed between groups for *K*_*m*_, CCT, and postoperative follow‐up time (*p* > 0.05), as shown in Table 1.

**TABLE 1:** Comparison of age and ocular parameters among different LT groups.

Variable	Total	Group 1 LT < 4.0 mm	Group 2 4.0 ≤ LT < 5.0 mm	Group 3 LT ≥ 5.0 mm	*p*‐value
Number (*n*)	431	104	221	106	NA
Age (years)	64.32 ± 9.74 (40.0, 85.0)	59.72 ± 9.57	64.59 ± 9.05	68.25 ± 9.48	< 0.001
AL (mm)	23.71 ± 1.69 (20.85, 30.27)	24.05 ± 1.82	23.66 ± 1.65	23.47 ± 1.58	0.036
*K*_*m*_ (D)	44.11 ± 1.51 (38.52, 48.09)	44.13 ± 1.25	44.16 ± 1.60	43.97 ± 1.57	0.594
ACD (mm)	3.06 ± 0.48 (1.84, 4.26)	3.53 ± 0.34	3.05 ± 0.38	2.65 ± 0.39	< 0.001
LT (mm)	4.50 ± 0.58 (3.23, 5.96)	3.74 ± 0.17	4.50 ± 0.27	5.24 ± 0.23	< 0.001
WTW (mm)	11.74 ± 0.40 (10.50, 13.00)	11.72 ± 0.38	11.70 ± 0.39	11.83 ± 0.43	0.020
CCT (μm)	543.68 ± 33.94 (417.00, 659.00)	544.32 ± 32.18	543.00 ± 33.82	544.48 ± 36.11	0.912
IOL power (D)	20.21 ± 4.34 (4.50, 32.00)	19.16 ± 4.53	20.26 ± 4.39	21.12 ± 3.84	0.004
ELP_error_ (mm)	−0.04 ± 0.43 (−1.75, 2.73)	−0.13 ± 0.50	−0.07 ± 0.38	0.13 ± 0.42	< 0.001
Follow‐up (weeks)	8.77 ± 6.01 (4, 24)	9.73 ± 7.02	8.42 ± 5.37	8.57 ± 6.16	0.172

*Note.* Data are presented as mean ± standard deviation (range). Km, mean keratometry; WTW, horizontal corneal diameter (white‐to‐white); ELPerror, the prediction error of effective lens position; follow‐up, postoperative follow‐up time (weeks); *p*‐values were calculated using one‐way ANOVA across the three lens thickness groups (*p* < 0.05 indicates statistical significance).

Abbreviations: AL, axial length; ACD, anterior chamber depth; LT, lens thickness; CCT, central corneal thickness; NA, not applicable.

### 3.2. LT on Postoperative Refractive Error

Statistically significant differences were observed for both PE (*F* = 25.742, *p* < 0.001) and ELP_error_ (*F* = 10.838, *p* < 0.001) across different LT groupings. LSD post hoc analysis revealed significant differences in PE and ELP_error_ parameters between Group 3 versus both Group 1 and Group 2 (*p* < 0.001). A statistically significant intergroup difference was only detected in the PE parameter between Group 1 and Group 2 (*p* = 0.036), as shown in Table 2.

**TABLE 2:** Post hoc pairwise comparisons with 95% CI.

	PE			ELP_error_		
Comparison	Mean diff (D)	95% CI	*p*‐value	Mean diff (mm)	95% CI	*p*‐value
G1 vs G2	−0.1037	[−0.2008, −0.0066]	0.036	−0.0530	[−0.1521, 0.0461]	0.294
G1 vs G3	−0.3894	[−0.5021, −0.2767]	< 0.001	−0.2512	[−0.3662, −0.1361]	< 0.001
G2 vs G3	−0.2857	[−0.3822, −0.1892]	< 0.001	−0.1982	[−0.2967, −0.0997]	< 0.001

*Note.* LSD (least significant difference) post hoc test was used. Mean Diff = mean difference between two groups. 95% CI = 95% confidence interval for the mean difference. PE overall ANOVA: *F* = 25.742, *p* < 0.001. ELPerror Overall ANOVA: *F* = 10.838, *p* < 0.001.

Subgroup analysis (Table 3) revealed that Group 1 and Group 2 had negative PE values, whereas Group 3 had positive PE values. Group 2 showed the lowest MAE (0.33 D) and MedAE (0.29 D), with the highest proportions of eyes within ± 0.50 D (78.3%) and ± 1.0 D (100.0%). However, Group 3 had the highest proportion of eyes within ± 0.25 D (44.3%). Group 1 showed the poorest performance across all evaluated metrics.

**TABLE 3:** Comparison of the predictive performance of different LT groups.

Variable	PE	MAE	MedAE	±0.25D	±0.5D	±1.0D
ALL	−0.08 ± 0.44	0.35	0.31	42.0%	75.6%	97.4%
Group 1	−0.23 ± 0.45	0.40	0.32	40.4%	72.1%	94.2%
Group 2	−0.13 ± 0.38	0.33	0.29	41.6%	78.3%	100.0%
Group 3	0.16 ± 0.45	0.37	0.29	44.3%	73.6%	95.3%

### 3.3. Relationship Between LT and PE

**FIGURE 1:**
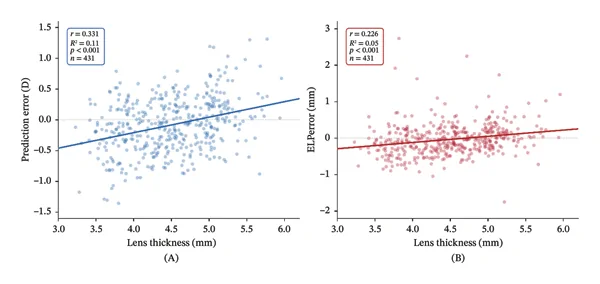
Linear relationship between LT and PE, ELP_error_. LT = lens thickness; ELPerror = the prediction error of effective lens position; PE = the postoperative refractive error; *r* = Pearson correlation coefficient. The figure demonstrates a significant positive linear correlation between LT values and both PE (A) and ELPerror (B) (*p* < 0.001), with *R* ^2^ values of 0.11 and 0.05, respectively.

Pearson correlation analysis revealed a significant positive correlation between LT and PE (*r* = 0.331, *p* < 0.001). As illustrated in Figure 1, as LT increased, the postoperative PE in patients gradually shifted from negative values (myopic state) to positive values (hyperopic state). Furthermore, when LT approached the population mean of healthy individuals, the PE value approached zero. Multiple linear regression analysis was performed with postoperative PE as the dependent variable and seven preoperative biometric parameters (age, AL, ACD, LT, *K*_*m*_, WTW, and CCT) as independent variables, based on a prespecified clinical rationale without variable selection. The model collectively explained 21.2% of the variance in postoperative PE (*R* ^2^ = 0.212, adjusted *R* ^2^ = 0.199, *F* (7, 423) = 16.301, *p* < 0.001; Durbin–Watson = 2.016). Among the seven predictors, LT emerged as the strongest independent predictor of PE (*β* = 0.490, *B* = 0.372, 95% CI [0.260, 0.485], *t* = 6.522, *p* < 0.001), followed by ACD (*β* = 0.217, *B* = 0.197, 95% CI [0.044, 0.350], *t* = 2.529, *p* = 0.012) and WTW (*β* = 0.130, *B* = 0.142, 95% CI [0.013, 0.272], *t* = 2.165, *p* = 0.031). The remaining variables—AL (*β* = 0.073, *p* = 0.244), *K*_*m*_ (*β* = −0.051, *p* = 0.409), age (*β* = −0.013, *p* = 0.793), and CCT (*β* = 0.018, *p* = 0.684)—did not reach statistical significance. The relative importance of the significant predictors was as follows: LT > ACD > WTW (Table 4).

**TABLE 4:** Multiple linear regression model for prediction error.

Variable	B	SE	*β*	*t*	*p*‐value	95% CI
(Constant)	−3.9240	1.5039	—	−2.609	0.0094	[−6.8801, −0.9679]
Age	−0.0006	0.0022	−0.0127	−0.262	0.7931	[−0.0049, 0.0037]
AL	0.0189	0.0162	0.0726	1.167	0.2440	[−0.0129, 0.0507]
ACD	0.1968	0.0778	0.2166	2.529	0.0118	[0.0439, 0.3497]
LT	0.3725	0.0571	0.4898	6.522	< 0.001	[0.2602, 0.4847]
*K*_*m*_	−0.0147	0.0178	−0.0508	−0.827	0.4089	[−0.0497, 0.0203]
WTW	0.1423	0.0657	0.1304	2.165	0.0309	[0.0131, 0.2715]
CCT	0.0002	0.0006	0.0183	0.407	0.6845	[−0.0009, 0.0014]

*Note:* *R* ^2^ = 0.2124, adjusted *R* ^2^ = 0.1994, *F* (7, 423) = 16.301, *p* < 0.001, *N* = 431. Durbin–Watson = 2.0161. AIC = 425.06, BIC = 457.59. *β* = standardized regression coefficient; *B* = unstandardized regression coefficient; 95% CI = 95% confidence interval for B.

Abbreviation: SE, standard error.

### 3.4. Correlation Analysis Between LT and ELP

A correlation analysis was conducted to evaluate the interrelationships among ELP_true_, ELP_predict_, ELP_error_, and LT. The results demonstrated no statistically significant correlation between ELP_true_ and LT (*r* = −0.003, *p* = 0.952). ELP_predict_ exhibited a weak negative correlation with LT (*r* = −0.179, *p* < 0.001), while ELP_error_ showed a weak positive correlation with LT (*r* = 0.226, *p* < 0.001). Specifically, as LT increased, the formula‐predicted ELP values transitioned from overestimation to underestimation, resulting in a gradual shift of ELP_error_ from negative to positive values. Notably, when LT approached the population mean, ELP_error_ approached zero, indicating improved predictive accuracy.

**FIGURE 2:**
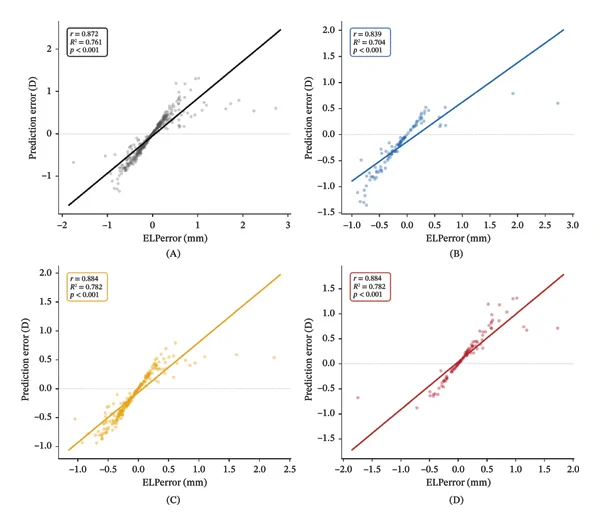
Linear relationship analysis between PE and ELP_error_ in different LT groups. ELPerror = the prediction error of effective lens position; PE = the postoperative refractive error; *r* = Pearson correlation coefficient. Both PE and ELPerror demonstrated a significant positive linear association in the full cohort (A) and stratified LT subgroups (B: Group 1, LT < 4.0 mm; C: Group 2, 4.0 ≤ LT < 5.0 mm; D: Group 3, LT ≥ 5.0 mm) (*p* < 0.001).

As illustrated in Figure 2 and Table 5, all LT subgroups exhibited strong linear relationships between PE and ELP_error_ (*p* < 0.001). Overall the analysis revealed that a 1‐mm increase in LT corresponded to a 0.17‐mm increase in ELP_error_ and a 0.251 D increase in PE. Subgroup analysis based on LT demonstrated that when the ELP_error_ was ± 1 mm, Group 1 exhibited PE change values of 0.620 D and 0.894 D; Group 2 showed PE change values of 0.803 D and 0.935 D; and Group 3 demonstrated PE change values of 0.988 D and 0.912 D.

**TABLE 5:** Linear relationship between PE and ELP_error_ in different LT groups.

Variable	*R* ^2^‐value	*p*‐value	Equation
ALL	0.761	< 0.001	PE = 0.882 × ELP_error_ −0.051
Group 1	0.704	< 0.001	PE = 0.757 × ELP_error_ −0.137
Group 2	0.782	< 0.001	PE = 0.868 × ELP_error_ −0.066
Group 3	0.782	< 0.001	PE = 0.950 × ELP_error_ +0.038

*Note.* *R* ^2^ = coefficient of determination. Group 1: LT < 4.0 mm; Group 2: 4.0 ≤ LT < 5.0 mm; Group 3: LT ≥ 5.0 mm. ALL represents the entire sample (*N* = 431). Group 1: *n* = 104; Group 2: *n* = 221; Group 3: *n* = 106. Linear regression was performed with PE as the dependent variable and ELPerror as the independent variable.

Abbreviations: PE, prediction error; ELP_error_, effective lens position error; LT, lens thickness.

### 3.5. Adjusted Analysis of LT Group Effect on Prediction Error

**FIGURE 3:**
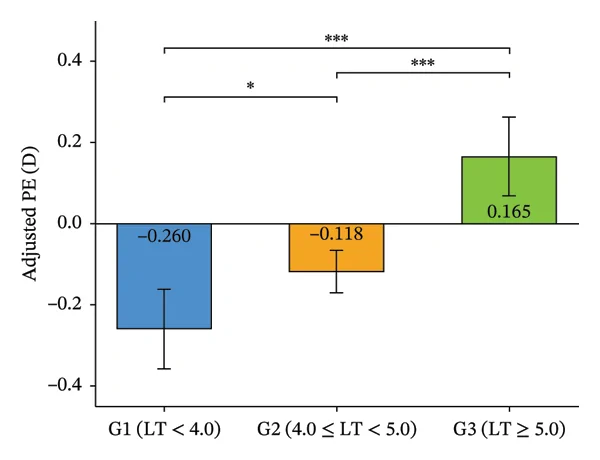
Adjusted prediction error by the LT group (ANCOVA). Adjusted least‐squares means of PE by the LT group, controlling for age, axial length (AL), anterior chamber depth (ACD), and white‐to‐white (WTW). Error bars represent 95% confidence intervals. The LT group effect remained significant after covariate adjustment (*F* = 14.960, *p* < 0.001, partial *η* ^2^ = 0.066). ^∗^ *p* < 0.05, ^∗∗∗^ *p* < 0.001 (Bonferroni‐corrected).

To address the potential confounding effects of baseline differences across LT groups, ANCOVA was performed with PE as the dependent variable, LT group as the fixed factor, and age, AL, ACD, and WTW as covariates. The LT group effect remained statistically significant after adjusting for these confounders (*F* = 14.960, *p* < 0.001, partial *η* ^2^ = 0.066; *R* ^2^ = 0.187, adjusted *R* ^2^ = 0.175). The estimated marginal means (adjusted for covariates) were −0.260 D (95% CI: −0.358, −0.162) for Group 1, −0.118 D (95% CI: −0.171, −0.066) for Group 2, and 0.165 D (95% CI: 0.068, 0.262) for Group 3. Bonferroni‐corrected pairwise comparisons revealed significant differences between all three groups (G1 vs G2: *p* = 0.040; G1 vs G3: *p* < 0.001; G2 vs G3: *p* < 0.001), confirming that the LT–PE association persists independently of age, AL, ACD, and WTW (detailed statistical results are provided in Supporting File 1; Figure 3).

## 4. Discussion

With the continuous refinement of IOL power calculation formulas, next‐generation formulas have incorporated multiple parameters, including LT, to further improve the accuracy of postoperative refraction [13]. However, the actual value of LT in enhancing the accuracy of postoperative refractive prediction remains inconsistent across studies, and its clinical significance is still debated [12, 14, 15]. This study systematically evaluated the association between LT and postoperative PE based on the SRK/T formula. The findings revealed that LT affects the estimation of ELP, leading to postoperative refractive errors, with the magnitude of error varying across different LT groups.

Compared with other modern IOL formulas, the SRK/T formula was selected for this study partly because it remains one of the most widely used IOL power calculation formulas in clinical practice worldwide [16], and it features a transparent and well‐defined ELP prediction algorithm. Additionally, since SRK/T does not incorporate LT in its ELP estimation, any systematic association between LT and PE can be directly attributed to the formula’s failure to account for this parameter—thereby directly revealing the clinical impact of LT on the accuracy of ELP prediction. Had we used formulas that already incorporate LT (e.g., Barrett Universal II, Kane, or Pearl‐DGS), this association would have been partially or completely obscured. However, this association is not unique to the SRK/T formula. Multiple studies have demonstrated [11, 13, 17] that as LT increases from extremely thin to extremely thick, the prediction error of modern IOL formulas—including Barrett Universal II, Kane, and Pearl‐DGS—consistently shifts from a myopic direction toward a hyperopic direction. These findings indicate that the systematic prediction error associated with extreme LT values persists even when using modern formulas that incorporate LT. This further underscores the clinical significance of including LT as a biometric parameter across different IOL power calculation formulas.

This study conducted a subgroup analysis based on LT, revealing statistically significant differences in age, AL, ACD, and WTW corneal diameter among LT subgroups. Patients with thicker LT exhibited biological characteristics such as advanced age, shorter AL, shallower ACD, and larger WTW, whereas patients with thinner LT exhibited younger age, longer AL, deeper ACD, and smaller WTW. These findings align with those of Meng et al. [18], who investigated thick LT. López‐de la Rosa et al. reported that demographic characteristics and certain ocular biometric parameters are associated with LT, and therefore confounding factors must be controlled for when analyzing LT; otherwise, the results may be severely biased [19]. Accordingly, in the present study, ANCOVA was performed adjusting for age, AL, ACD, and WTW. The LT–PE association remained significant after adjustment, with Bonferroni‐corrected pairwise comparisons confirming significant differences across all three LT groups, indicating that the influence of LT on PE is independent of these confounding biometric parameters.

Further subgroup analysis showed that the normal LT group achieved superior IOL power prediction accuracy, with reduced dispersion of prediction errors, whereas the thin LT group exhibited the poorest predictive performance—primarily attributable to a higher proportion of patients with long AL in this subgroup. As noted by Chen et al. [20], longer AL correlates with lens flattening, resulting in reduced LT. Additionally, eyes with elongated axial dimensions often exhibit posterior staphyloma and complex anatomical changes in ocular biometry [21], all of which synergistically compromise the accuracy of refractive prediction in this population. Zhang et al. [22] reported that the SRK/T formula had the lowest prediction accuracy in long AL eyes (> 24.5 mm), significantly inferior to other formulas such as Barrett Universal II. Correlation analysis revealed that increasing LT gradually reduced myopic shift error while progressively amplifying hyperopic shift error. Specifically, the thin LT group exhibited more pronounced myopic shift, whereas the thick LT group demonstrated greater hyperopic shift. This conclusion aligns with Ambade et al. [23], who reported that refractive errors calculated using the SRK/T formula exhibit mild hyperopic drift with increasing LT. Furthermore, other studies have demonstrated that PE calculated by different formulas exhibits similar linearly escalating trends as LT increases [11, 17].

In clinical practice, postoperative PE is commonly attributed to longitudinal displacement of the IOL along the optical axis [24]. Specifically, myopic refractive shifts are typically associated with anterior IOL displacement, whereas hyperopic shifts are linked to posterior displacement. Chen et al. utilized preoperative and postoperative anterior segment swept‐source optical coherence tomography (SS‐OCT) to measure the actual IOL position changes and found significant anterior IOL displacement in the thick LT group, resulting in myopic refractive errors [25]. This finding contradicts our observation of greater hyperopic refractive errors in patients with thick LT postsurgery. By leveraging the open‐source framework of the SRK/T formula, we further incorporated ELP_error_ values to elucidate the mechanisms underlying PE variations caused by differing LT values. Unlike previous studies that measured the “true” ELP (ELP_true_) using anatomical imaging modalities such as AS‐OCT or ultrasound biomicroscopy (UBM), we derived the ELP_true_ by back‐calculation using the SRK/T formula based on the postoperative SE and the power of the implanted IOL. Consequently, ELP_error_ (ELP_true_ − ELP_predicted_) represents the discrepancy between the formula‐predicted ELP and the ELP that would have been required to achieve the actual postoperative refraction. Our analysis revealed a significant negative correlation between LT and predicted ELP. For patients with LT < 4.0 mm, the SRK/T formula overestimates postoperative ELP, leading to a predicted anterior IOL position and consequent myopic refractive error. Conversely, in patients with LT ≥ 5.0 mm, the formula underestimates ELP, resulting in a predicted posterior IOL position and hyperopic refractive error. We propose that formula‐derived ELP errors do not fully equate to direct axial IOL displacement measurements, as they may also reflect structural changes in other intraocular components. Studies indicate that early capsular hydration‐induced expansion often causes IOL anterior displacement (myopic shift), while long‐term capsular fibrosis and contraction induce posterior displacement (hyperopic shift) [26]. This dynamic nature of postoperative anatomical changes further complicates refractive prediction. Thus, we conclude that ELP estimation deviations in IOL formulas are the primary contributors to postoperative PE, and optimizing ELP prediction accuracy can enhance postoperative refractive outcomes [27]. Furthermore, LT emerged as the strongest independent predictor of postoperative PE, a finding consistent with Chen et al. [12], who identified LT as a critical prognostic factor for PE in modern IOL formulas (e.g., Kane, Hill‐RBF 3.0, BUII, EVO, and Pearl‐DGS).

Quantitative analysis of ELP_error_, LT, and PE relationships demonstrated that each 1‐mm increase in LT correlates with a 0.17‐mm increase in ELP_error_ and a 0.251 D increase in PE. However, the magnitude of PE variation per ELP_error_ differs across LT subgroups. Subgroup analysis revealed that patients with thick LT exhibit significantly higher PE values for equivalent ELP errors compared to other groups. This phenomenon may stem from anatomical associations of thick LT, including shallow anterior chambers, narrow angles (risk factors for angle‐closure glaucoma) [28], zonular laxity or partial lens dislocation risks [8], and larger capsular diameters and volumes [29]. These structural alterations likely amplify PE magnitudes.

This study has several limitations. First, it included single‐center data with limited sample sizes of eyes exhibiting abnormal LT extremes, necessitating multicenter validation. Second, multiple IOL types were included without subgroup stratification; although monofocal IOLs were calculated using the SRK/T formula and other IOL types using both the Barrett Universal II and SRK/T formulas, the potential influence of IOL model heterogeneity on the results cannot be entirely excluded. Third, the present analysis focused primarily on the SRK/T formula, which limits the generalizability of the findings. Future studies will incorporate multiple modern IOL formulas for more comprehensive comparative analyses. Fourth, ELP_true_ in this study was derived from postoperative refraction, and the strong association between ELP_error_ and PE is partly expected, potentially leading to an overestimation of the true strength of this relationship.

In conclusion, our findings demonstrated that increased deviation of LT from the mean results in larger ELP prediction errors and greater postoperative refractive error—specifically, myopia in thin‐lens eyes and hyperopia in thick‐lens eyes. These results highlight the need to account for deviations in LT and refine ELP estimation to enhance the accuracy of IOL power calculations.

**Nomenclature**
LT: lens thickness
PE: prediction error
MAE: mean absolute error
MedAE: median absolute error
ELP: effective lens position
AL: axial length
K_m_
: mean keratometry
ACD: anterior chamber depth
IOL: intraocular lens
BCVA: best‐corrected visual acuity
WTW: white‐to‐white
CCT: central corneal thickness
SE: spherical equivalent
ANOVA: one‐way analysis of variance
ANCOVA: analysis of covariance
ELP_predict_
: predicted effective lens position
ELP_true_
: true effective lens position
ELP_error_
: effective lens position error
SD: standard deviation
IQR: interquartile range
ULIB: User Group for Laser Interference Biometry
SS‐OCT: swept‐source optical coherence tomography
BUII: Barrett Universal II
CI: confidence interval
UBM: ultrasound biomicroscopy
AS‐OCT: anterior segment OCT.

## Author Contributions

Conceptualization: Yong Wang and Haifeng Jiang. Data curation: Tao Liu, Haifeng Jiang, Yanxia Tong, Zhanpeng Zhu, and Lihan Luo. Formal analysis: Haifeng Jiang and Yanxia Tong. Funding acquisition: Yong Wang. Investigation: Haifeng Jiang, Zhanpeng Zhu, and Tao Liu. Methodology: Yong Wang and Haifeng Jiang. Project administration: Yong Wang. Resources: Yong Wang. Software: Haifeng Jiang and Yanxia Tong. Supervision: Yong Wang. Validation: Tao Liu, Haifeng Jiang, and Lihan Luo. Visualization: Haifeng Jiang. Writing–original draft: Haifeng Jiang. Writing–review and editing: Tao Liu and Yong Wang.

## Funding

This study was supported by the Hubei Provincial Health Commission Science and Technology Project (Grant No. WJ2025M093), Science Research Foundation of Aier Eye Hospital Group (Grant No. AGK2302D09), Xiangjiang Public Welfare Foundation Technology Innovation and Application Development Project (KY24013).

## Disclosure

All authors read and approved the final manuscript.

## Ethics Statement

The study obtained approval from the Ethics Committee of Aier Eye Hospital of Wuhan University (China), and all study procedures complied with the Declaration of Helsinki (Approval NO: 2024IRBKY112507). Written informed consent for the use of clinical data was routinely obtained from each patient before cataract surgery.

## Conflicts of Interest

The authors declare no conflicts of interest.

## Supporting Information

Additional supporting information can be found online in the Supporting Information section.

## Supporting information


**Supporting Information** ANCOVA Statistical Results: ANCOVA was performed with PE as the dependent variable, LT group as the fixed factor, and age, AL, ACD, and WTW as covariates.

## Data Availability

The datasets generated and analyzed during the current study are not publicly available due to patient privacy concerns but are available from the corresponding author on reasonable request.

## References

[1] Khoramnia R. , Auffarth G. , Łabuz G. , Pettit G. , and Suryakumar R. , Refractive Outcomes After Cataract Surgery, Diagnostics. (2022) 12, no. 2, 10.3390/diagnostics12020243. PMC8870878 35204334

[2] Cicinelli M. V. , Buchan J. C. , Nicholson M. , Varadaraj V. , and Khanna R. C. , Cataracts, The Lancet. (2023) 401, no. 10374, 377–389, 10.1016/S0140-6736(22)01839-6. 36565712

[3] Lundström M. , Dickman M. , Henry Y. et al., Risk Factors for Refractive Error After Cataract Surgery: Analysis of 282 811 Cataract Extractions Reported to the European Registry of Quality Outcomes for Cataract and Refractive Surgery, Journal of Cataract & Refractive Surgery. (2018) 44, no. 4, 447–452, 10.1016/j.jcrs.2018.01.031. 29685779

[4] Gaurisankar Z. S. , van Rijn G. A. , Lima J. E. E. et al., Correlations Between Ocular Biometrics and Refractive Error: A Systematic Review and Meta-Analysis, Acta Ophthalmologica. (2019) 97, no. 8, 735–743, 10.1111/aos.14208. 31386806

[5] Donaldson P. J. , Grey A. C. , Maceo Heilman B. , Lim J. C. , and Vaghefi E. , The Physiological Optics of the Lens, Progress in Retinal and Eye Research. (2017) 56, e1–e24, 10.1016/j.preteyeres.2016.09.002. 27639549

[6] Nishida S. , Inomata Y. , and Hirata A. , Risk Factors for Postoperative Refractive Error in New-Generation Intraocular Lens Calculation Formulas, Clinical Ophthalmology. (2024) 18, 2253–2259, 10.2147/OPTH.S471393. PMC11328866 39157049

[7] Retzlaff J. A. , Sanders D. R. , and Kraff M. C. , Development of the SRK/T Intraocular Lens Implant Power Calculation Formula, Journal of Cataract & Refractive Surgery. (1990) 16, no. 3, 333–340, 10.1016/s0886-3350(13)80705-5. 2355321

[8] Yang J. , Fan Q. , Chen J. et al., The Efficacy of Lens Removal plus IOL Implantation for the Treatment of Spherophakia with Secondary Glaucoma, British Journal of Ophthalmology. (2016) 100, no. 8, 1087–1092, 10.1136/bjophthalmol-2015-307298. 26608028

[9] Gupta S. , Warjri G. , and Gupta V. , Ɛ’ Sign: A Clinical Clue to the Diagnosis of Microspherophakia, Eye. (2020) 34, no. 3, 597–599, 10.1038/s41433-019-0544-6. PMC7042234 31391543

[10] Zhu X. , He W. , Sun X. , Dai J. , and Lu Y. , Fixation Stability and Refractive Error After Cataract Surgery in Highly Myopic Eyes, American Journal of Ophthalmology. (2016) 169, 89–94, 10.1016/j.ajo.2016.06.022. 27325397

[11] Melles R. B. , Holladay J. T. , and Chang W. J. , Accuracy of Intraocular Lens Calculation Formulas, Ophthalmology. (2018) 125, no. 2, 169–178, 10.1016/j.ophtha.2017.08.027. 28951074

[12] Chen Y. , Fang Y. , Zhao J. , He W. , Ma B. , and Zhu X. , Influence of Lens Thickness on Accuracy of Kane, Hill-RBF 3.0, Barrett Universal II, Emmetropia Verifying Optical, and Pearl-DGS Formulas in Eyes With Nonhigh Myopia and High Myopia, Current Eye Research. (2024) 49, no. 6, 605–614, 10.1080/02713683.2024.2316717. 38363071

[13] Hipólito-Fernandes D. , Luís M. E. , Serras-Pereira R. et al., Anterior Chamber Depth, Lens Thickness and Intraocular Lens Calculation Formula Accuracy: Nine Formulas Comparison, British Journal of Ophthalmology. (2022) 106, no. 3, 349–355, 10.1136/bjophthalmol-2020-317822. 33229347

[14] Plat J. , Hoa D. , Mura F. et al., Clinical and Biometric Determinants of Actual Lens Position After Cataract Surgery, Journal of Cataract & Refractive Surgery. (2017) 43, no. 2, 195–200, 10.1016/j.jcrs.2016.11.043. 28366366

[15] Taroni L. , Hoffer K. J. , Lupardi E. , Barboni P. , and Savini G. , Accuracy of New Intraocular Lens Power Calculation Formulas: A Lens Thickness Study, Journal of Refractive Surgery. (2021) 37, no. 3, 202–206, 10.3928/1081597X-20210104-01. 34038301

[16] Can İ. , Takmaz T. , Özdamar A. et al., Evaluation of the Cataract Surgery 2018 Survey in Terms of Achieving Refractive Cataract Surgery Targets, Turk J Ophthalmol. (2021) 51, no. 1, 7–18, 10.4274/tjo.galenos.2020.46020. PMC7931655 33631897

[17] Yuan H. , Zhang J. , Han X. et al., Accuracy of 11 Intraocular Lens Calculation Formulas in Shallow Anterior Chamber Eyes, Acta Ophthalmologica. (2024) 102, no. 5, e705–e711, 10.1111/aos.16654. 38334238

[18] Meng J. , Wei L. , He W. , Qi J. , Lu Y. , and Zhu X. , Lens Thickness and Associated Ocular Biometric Factors Among Cataract Patients in Shanghai, Eye and Vis. (2021) 8, no. 1, 10.1186/s40662-021-00245-3. PMC8165789 34053465

[19] López-de la Rosa A. , Díez-Montero C. , Martínez-Plaza E. , López-Miguel A. , and Maldonado M. J. , Senile Cataract Formation Does Not Affect Crystalline Lens Thickness, Ophthalmol Ther. (2024) 13, no. 3, 819–830, 10.1007/s40123-024-00882-6. PMC10853159 38273047

[20] Chen C. , Meng J. , Cheng K. et al., Spatial and Morphologic Features of Lenses With Different Axial Lengths in Cataract Patients: A swept-source Optical Coherence Tomography-Based Study, BMC Ophthalmology. (2024) 24, no. 1, 10.1186/s12886-024-03813-y. PMC11660780 39702138

[21] Olsen T. , Intraocular Lens Power Calculation Errors in Long Eyes, Journal of Cataract & Refractive Surgery. (2012) 38, no. 4, 733–734, 10.1016/j.jcrs.2012.02.003. 22440455

[22] Zhang C. , Dai G. , Pazo E. E. et al., Accuracy of Intraocular Lens Calculation Formulas in Cataract Patients With Steep Corneal Curvature, PLoS One. (2020) 15, no. 11, 10.1371/journal.pone.0241630. PMC7678954 33216749

[23] Ambade A. K. , Ambade R. A. , and Raje D. V. , Refractive Prediction by Various Intraocular Lens Formulas Using Optical Biometry and Effect of Ocular Parameters on Their Accuracy, Indian Journal of Ophthalmology. (2022) 70, no. 1, 118–123, 10.4103/ijo.IJO_31_21. PMC8917615 34937222

[24] Erickson P. , Effects of Intraocular Lens Position Errors on Postoperative Refractive Error, Journal of Cataract & Refractive Surgery. (1990) 16, no. 3, 305–311, 10.1016/s0886-3350(13)80699-2. 2355315

[25] Chen C. , Zhou J. , Cheng K. et al., Lens Position Change Following Cataract Surgery in Eyes with Thick Lenses: An SS-OCT Based Study, British Journal of Ophthalmology. (2025) 109, no. 10, 1120–1125, 10.1136/bjo-2025-327414. 40461064

[26] Kamath G. G. , Prasad S. , Patwala Y. J. , and Phillips R. P. , Postoperative Myopia With Subsequent Hyperopic Shift After Phacoemulsification and Multifocal IOL Implantation, Journal of Cataract & Refractive Surgery. (2001) 27, no. 5, 651–652, 10.1016/s0886-3350(01)00827-6. 11405203

[27] Li T. , Stein J. , and Nallasamy N. , AI-powered Effective Lens Position Prediction Improves the Accuracy of Existing Lens Formulas, British Journal of Ophthalmology. (2022) 106, no. 9, 1222–1226, 10.1136/bjophthalmol-2020-318321. PMC9411905 33836989

[28] Miao A. , Yang F. , Chen T. et al., Lens Thickness-to-Anterior Chamber Depth Ratio: A Biometric Determinant of Chamber Angle Width in Han Chinese Cataract Patients, Investigative Ophthalmology & Visual Science. (2025) 66, no. 3, 10.1167/iovs.66.3.42. PMC11932420 40111355

[29] Waring G. O. , Chang D. H. , Rocha K. M. , Gouvea L. , and Penatti R. , Correlation of Intraoperative Optical Coherence Tomography of Crystalline Lens Diameter, Thickness, and Volume With Biometry and Age, American Journal of Ophthalmology. (2021) 225, 147–156, 10.1016/j.ajo.2020.12.021. 33385370

