# Supplementary material for: Association Between Lens Thickness and SRK/T Refractive Prediction Error After Cataract Surgery: A Single‐Center Retrospective Cohort Study
Source: J Ophthalmol. 2026 Sep 22;2026:4944226. doi: 10.1155/joph/4944226 (PMC13595810; doi:10.1155/joph/4944226)
Supplement: Supplementary file 1 [file JOPH-2026-4944226-s001.docx]

**Supplementary File 1: ANCOVA Statistical Results**

ANCOVA was performed with PE as the dependent variable, LT group as the fixed factor, and age, AL, ACD, and WTW as covariates.

**Table S2-1. Tests of Between-Subjects Effects (ANCOVA)**

| **Source** | **Type III SS** | **df** | **Mean Square** | **F** | **p-value** | **Partial η²** |
| --- | --- | --- | --- | --- | --- | --- |
| Corrected Model | 15.464 | 6 | 2.577 | 16.234 | < 0.001 | 0.187 |
| Intercept | 4.167 | 1 | 4.167 | 26.246 | < 0.001 | 0.058 |
| Age | 0.000 | 1 | 0.000 | 0.001 | 0.970 | 0.000 |
| AL | 0.986 | 1 | 0.986 | 6.212 | 0.013 | 0.014 |
| ACD | 0.063 | 1 | 0.063 | 0.394 | 0.531 | 0.001 |
| WTW | 2.130 | 1 | 2.130 | 13.415 | < 0.001 | 0.031 |
| LT Group | 4.750 | 2 | 2.375 | 14.960 | < 0.001 | 0.066 |
| Error | 67.313 | 424 | 0.159 | — | — | — |
| Total | 85.744 | 431 | — | — | — | — |
| Corrected Total | 82.777 | 430 | — | — | — | — |

Dependent variable: SRK/T prediction error (PE). R² = 0.187; Adjusted R² = 0.175.

**Table S2-2. Parameter Estimates (reference: Group 3)**

| **Parameter** | **B** | **SE** | **t** | **p-value** | **95% CI** | **Partial η²** |
| --- | --- | --- | --- | --- | --- | --- |
| Intercept | −3.163 | 0.680 | −4.654 | < 0.001 | [−4.499, −1.827] | 0.049 |
| Age | 8.199E−5 | 0.002 | 0.037 | 0.970 | [−0.004, 0.004] | 0.000 |
| AL | 0.036 | 0.014 | 2.492 | 0.013 | [0.008, 0.064] | 0.014 |
| ACD | 0.042 | 0.066 | 0.628 | 0.531 | [−0.089, 0.172] | 0.001 |
| WTW | 0.199 | 0.054 | 3.663 | < 0.001 | [0.092, 0.306] | 0.031 |
| [LT Group = 1] | −0.424 | 0.083 | −5.133 | < 0.001 | [−0.587, −0.262] | 0.059 |
| [LT Group = 2] | −0.283 | 0.056 | −5.032 | < 0.001 | [−0.394, −0.172] | 0.056 |
| [LT Group = 3] | 0ᵃ | — | — | — | — | — |

ᵃ This parameter is set to zero because it is redundant (reference category). Covariates evaluated at: AL = 23.708 mm, ACD = 3.065 mm, WTW = 11.741 mm, Age = 64.318 years.

**Table S2-3. Estimated Marginal Means of PE by LT Group**

| **LT Group** | **Adjusted Mean (D)** | **SE** | **95% CI** |
| --- | --- | --- | --- |
| Group 1 (LT < 4.0 mm) | −0.260 | 0.050 | [−0.358, −0.162] |
| Group 2 (4.0 ≤ LT < 5.0 mm) | −0.118 | 0.027 | [−0.171, −0.066] |
| Group 3 (LT ≥ 5.0 mm) | 0.165 | 0.049 | [0.068, 0.262] |

Covariates evaluated at: AL = 23.708 mm, ACD = 3.065 mm, WTW = 11.741 mm, Age = 64.318 years.

**Table S2-4. Pairwise Comparisons of Adjusted PE Between LT Groups (Bonferroni-corrected)**

| **Comparison** | **Mean Difference (D)** | **SE** | **p-value** | **95% CI** |
| --- | --- | --- | --- | --- |
| G1 vs G2 | −0.141 | 0.057 | 0.040 | [−0.278, −0.005] |
| G1 vs G3 | −0.424 | 0.083 | < 0.001 | [−0.623, −0.226] |
| G2 vs G3 | −0.283 | 0.056 | < 0.001 | [−0.418, −0.148] |

Bonferroni-corrected pairwise comparisons. All three group comparisons were statistically significant.
